# Measles vaccination in humanitarian emergencies: a review of recent practice

**DOI:** 10.1186/1752-1505-5-21

**Published:** 2011-09-26

**Authors:** Rebecca F Grais, Peter Strebel, Peter Mala, John Watson, Robin Nandy, Michelle Gayer

**Affiliations:** 1 Epicentre, 8 rue Saint Sabin, Paris 75011, France; 2 World Health Organization, Avenue Appia 20, 1211 Geneva 27, Switzerland; 3 UNICEF, Health Section, Program Division, 3 United Nations Plaza, New York, New York 10017, USA

## Abstract

**Background:**

The health needs of children and adolescents in humanitarian emergencies are critical to the success of relief efforts and reduction in mortality. Measles has been one of the major causes of child deaths in humanitarian emergencies and further contributes to mortality by exacerbating malnutrition and vitamin A deficiency. Here, we review measles vaccination activities in humanitarian emergencies as documented in published literature. Our main interest was to review the available evidence focusing on the target age range for mass vaccination campaigns either in response to a humanitarian emergency or in response to an outbreak of measles in a humanitarian context to determine whether the current guidance required revision based on recent experience.

**Methods:**

We searched the published literature for articles published from January 1, 1998 to January 1, 2010 reporting on measles in emergencies. As definitions and concepts of emergencies vary and have changed over time, we chose to consider any context where an application for either a Consolidated Appeals Process or a Flash Appeal to the UN Central Emergency Revolving Fund (CERF) occurred during the period examined. We included publications from countries irrespective of their progress in measles control as humanitarian emergencies may occur in any of these contexts and as such, guidance applies irrespective of measles control goals.

**Results:**

Of the few well-documented epidemic descriptions in humanitarian emergencies, the age range of cases is not limited to under 5 year olds. Combining all data, both from preventive and outbreak response interventions, about 59% of cases in reports with sufficient data reviewed here remain in children under 5, 18% in 5-15 and 2% above 15 years. In instances where interventions targeted a reduced age range, several reports concluded that the age range should have been extended to 15 years, given that a significant proportion of cases occurred beyond 5 years of age.

**Conclusions:**

Measles outbreaks continue to occur in humanitarian emergencies due to low levels of pre-existing population immunity. According to available published information, cases continue to occur in children over age 5. Preventing cases in older age groups may prevent younger children from becoming infected and reduce mortality in both younger and older age groups.

## Background

Humanitarian emergencies occur in situations of conflict, war or civil disturbance, natural disasters, food insecurity or other crises resulting in disruptions that overwhelm national capacities and require international assistance [1]. The health needs of children and adolescents in humanitarian emergencies are critical to the success of relief efforts and reduction in mortality. Measles has been one of the major causes of child deaths in humanitarian emergencies and further contributes to mortality by exacerbating malnutrition and vitamin A deficiency. Many deaths attributed to diarrheal disease and pneumonia may also be associated with measles. In the past, measles case-fatality ratios in children in humanitarian emergencies have been as high as 20-30% [2]. During a famine in Ethiopia in 2000, measles alone or in combination with wasting accounted for 22% of 159 deaths among children under 5 years of age and 17% of 72 deaths among children 5-14 years [3].

Progress in global measles control has resulted in much higher population immunity in most parts of the world. Consequently, there has been a 78% reduction in measles mortality, from an estimated 733,000 deaths in 2000 to 164,000 deaths in 2008 [4]. Although outbreaks of measles are far less likely in many regions, interruption of measles virus transmission requires a high level of population immunity (> 90%) and measles outbreaks continue to occur in populations where such high levels of immunity cannot be maintained. Humanitarian emergencies often occur in populations with low levels of immunity, given long-term disruption of routine vaccination programs, poor infrastructure and access to health services, and therefore an increased risk of measles epidemics with consequent mortality.

Although preventive mass measles vaccination in emergency settings has not been the subject of controversy, and in fact is a part of standard international guidance to prevent outbreaks from occurring, to date there has not been a review of these interventions. Present guidance for humanitarian emergencies is largely based on a model of humanitarian relief, which is focused on camps sheltering refugees or internally displaced persons. These camps were often overcrowded, with high risk of epidemic-prone diseases such as measles, particularly during the acute phase of the crisis. Preventive mass vaccination of a targeted age group aims to reduce the risk of epidemics. However, the nature of humanitarian emergencies has changed over the past decades with increasing numbers of displaced persons and refugees now residing in urban environments and dispersed among host communities rather than just in camps [5]. Further, the coexistence of crises of differing nature and intensity in the same region renders defining the beginning and end of humanitarian crises difficult, if not irrelevant.

Measles epidemic risk may be more closely related to the characteristics of the affected population prior to the emergency, than to the precipitating event. It is thus important to consider that countries are in different stages of measles control. The Americas have seen the elimination of indigenous measles since 2002 while several other WHO regions (EURO, EMRO, WPRO and AFRO) have declared elimination goals, and SEARO region currently has a mortality reduction goal.

Our goal was to revisit the *WHO-UNICEF Joint Statement on Reducing Measles Mortality in Emergencies *[6] and the *Sphere Project Humanitarian Charter and Minimum Standards in Disaster Response *[7]. Taking into consideration the changing epidemiological landscape of measles and progress in measles control, as well as the changing nature of humanitarian emergencies, may suggest that current guidance may need to be updated. During the acute phase of an emergency, current guidance recommends a swift preventive mass vaccination campaign, along with vitamin A supplementation including all children from 6 months through 14 years of age. The WHO/UNICEF statement adds a contingency that at a minimum, children from 6 months through 4 years of age must be immunized.

Here, we review measles vaccination activities in humanitarian emergencies as documented in published literature. Our main interest was to review the available evidence focusing on the target age range for mass vaccination campaigns either in response to a humanitarian emergency or in response to an outbreak of measles in a humanitarian context to determine whether the current guidance required revision based on recent experience.

## Methods

We searched PubMed/MEDLINE, EMBASE, Latin American and Caribbean Center on Health Sciences Information (LILACS), Index Medicus for the Eastern Mediterranean Region (IMEMR) and African Index Medicus (AIM) for articles published from January 1, 1998 to January 1, 2010 in English, French, Italian, Portuguese or Spanish. We used the key words "measles" AND ("outbreak" OR "outbreaks" OR "epidemic" OR "epidemics" OR "emergency" OR "emergencies"). We selected 1998 as this was the first revision of the SPHERE guideline, although it was updated in 2004. The results of the above search were reviewed to identify and remove articles that did not report on measles in humans. Full-text was then obtained, reviewed by two reviewers, and independently categorized as "relevant" or "not relevant." Bibliographies of papers were also reviewed for additional citations. Any discrepancy between reviewers with regard to the relevancy of papers reviewed was resolved through discussion.

Any article that mentioned: i) a measles outbreak; ii) described vaccination coverage either before and/or after an outbreak; iii) a vaccination intervention (whether or not it was implemented); and iv) occurred in a humanitarian emergency defined here as a country that submitted and received a Consolidated Appeals Process (CAP) or Flash appeal to the UN Central Emergency Revolving Fund (CERF) between January 1, 1998 and January 1, 2010 were considered "relevant". A vaccination intervention was considered to be any vaccination intervention with measles-containing vaccine beyond routine services that are normally available at healthcare facilities.

As definitions and concepts of emergencies vary and have changed over time, we chose to consider any context where an application for either a CAP or a Flash Appeal occurred during the period examined. The CAP process brings aid organizations together to jointly plan, coordinate, implement and monitor their response to humanitarian emergencies, and to appeal for funds. CAP appeals occur when there is an acute humanitarian need caused by a conflict or a natural disaster; when the government is either unable or unwilling to address the humanitarian need and/or when a single agency cannot cover all the needs and additional support is required [8]. A Flash Appeal (Flash) is a tool for structuring a coordinated humanitarian response for the first three to six months of an emergency for interventions within the time frame of the CAP. Both appeals are coordinated by OCHA (United Nations Office for the Coordination of Humanitarian Affairs) with participation from NGOs and UN agencies. Although the CAP/Flash appeal may not have occurred the year of the report, we considered these contexts more vulnerable to measles outbreaks and therefore included reports of measles outbreaks between January 1, 1998 and January 1, 2010 in countries that appealed for aid anytime during this period [9]. We included publications from countries irrespective of their progress in measles control as humanitarian emergencies may occur in any of these contexts and as such, guidance applies irrespective of measles control goals.

## Results

We identified a total of 1267 articles through our search strategy. Of these 239 mentioned a measles epidemic occurring between 1998 and 2009. We were able to obtain all of these papers. However, of these 239 papers, only 39 (14%) actually reported on outbreaks occurring in crises in countries where CAP/Flash appeals occurred. The 39 papers identified described a total of 37 outbreaks, in 29 (78%) of which a measles mass vaccination intervention was mentioned as having been used. Upon further review, only 25 papers were retained. Those 14 papers discarded reported either on mathematical models of potential interventions or reported on epidemics occurring outside of the time frame but with delayed publication or in one case on an epidemic in a hospital.

For each of these reports, some covering an outbreak in the same country, we attempted to determine objectively the impact of the measles vaccination intervention as it pertains to age range based on the data provided. Table 1 describes the epidemiologic characteristics of the reviewed reports classified by region to provide context on measles control. Table 2 includes details on the mass vaccination intervention noting in particular the time to the response (where reported) and if there was evidence of an impact.

**Table 1:** Epidemiological Characteristics of Reviewed Outbreaks

Region/ Country^REF^	Flash/CAP/Years	Dates of Outbreak	Scope of outbreak (size)	Reported Cases	Age of Cases	Incidence per 100,000	Vaccination Coverage of Population	Vaccination Status of Cases
**AMERICAS**								

Bolivia [9]	2004, 2007, 2008	1998-2000	Nationwide (8 million)	2567	55% < 5 y ≈18% 5-14 y	32	1995-1997 < 90%	N/A

Haiti [10]	2003, 2005, 2007, 2008, 2010	3/00-9/01	Nationwide (6.8 million)	1149	N/A	14.1	1995-1999: 47% in 1 y olds	N/A

Colombia [11]	2003	1-7/02	10/33 departments	68	65% 1-4 y	5.5	80% < 1 y (2000) 91% < 1 y (2001)	N/A

Colombia [12]		2-3/02	3 departments (subset of Colombia o/b above)	9	55% < 5 y 45% 5-15 y	N/A	66-127% in affected municipalities	N/A

**ASIA**								

Afghanistan [15]	1999-2003 2006-2010	2001	Nationwide	8762	62% 0-4 y 29% 5-9 y 9% > 10 y	N/A	62-90%	N/A

Afghanistan [16]		2000	7 of 30 provinces	thousands	N/A	**N/A**	N/A	N/A

Afghanistan [17]		2001	Nationwide (362 sentinel sites, 12.5 million 6 m-12 y)	8762	62% 0-4 y 33% 5-12 y	70 (for 6 m - 12 y)	40-47%	N/A

India [13]	2005 (Indian Ocean)	Aug 08 to Mar 09	Rural (362072, 148540 children < 15 y)	1811	N/A	N/A	N/A	N/A

India [14]	2005 (Indian Ocean)	Dec 04-Jan 05	Coastal area (87284 - 8803 < 5 y)	30 in non-affected villages 71 in tsunami affected villages	(non-affected) 36.7% < 5 y 5 y < = 60% < 15 y 3.3% > = 15 y (affected) 43.7% < 5 y 5 y < = 56.3% < 15 y 0% > 15 y	1.7 in non-affected 1.3 in affected	Estimated: 95%	3%

Sri Lanka [19]	2002, 2003, 2006-2008	10/99 - 6/00	Nationwide (19 million)	15250 suspected 4611 confirmed	15% < 5 y 32% 5-14 y	24 ^(4611/19 M)^	90%	40% (of 3728 evaluated)

Laos [20,1]	2009	3/1999 - 3/2000	Nationwide (5 million); 4 villages subset (2871)	2634 nationally, 185 in 4 villages	57% > 5 y 40% 5-13 y (subsample of 185 in 4 villages)	53 (nationally) 6443 (4 village subset)	68% on avg in previous 4 y	35% (subsample of 185 in 4 villages) VE = 68%

**AFRICA**								

Niger [21]	2005	2003 Nationally, 1/1 - 15/4 in Mirriah District	Nationwide (12.5 million); Mirrah district (677,885)	50138 Nationally, 8817 Mirriah district	75% < 5 y 20% 5-14 y	400 Nationwide 1300 in Mirriah district	25-91% in past decade	12.3%

Kenya [28,1,2]	2001, 2006, 2008	7-11/1998	2 hospitals	1000	75% > 4 y	N/A	70-93%	39% (VE = 84%)

Tanzania [24]	1999-2001 Refugees from Burundi	3-5/2001	4 refugee camps in Kibondo District (170500)	1062	21% < 9 m 27% 9 m-5 y 31% 6-15 y	623	95%	82% 9 m-5 y 27% 6-15 y

Ethiopia [25]	2000-3, 2006-7	1-7/2000	Gode District, Ethiopia	N/A	N/A	N/A	57% (in 9-36 m)	3% (9-36 m)

Mozambique [29,1,3]	2000, 2001, 2003, 2007	Multiple 1998-2001	Nationwide (16 million)	Not clear, about 35-40,000	Varied greatly 30-85% 0-59 m	N/A	67-100%	N/A

Niger [21-23]	2005	2003-2004	Niamey (surveyed = 26795)	1024	82% < 5 y 3.5% > 15 y	N/A	70.9%	37.3%

Chad [23]	2004-2010	2004-2005	Ndjamena(surveyed = 21812)	745	70% < 5 y 4.4% > 15 y	N/A	33%	70%

South Africa [30,1,2,4]	2003, 2008	July 03-may 05	Johannesburg and rural (Oliver Tambo District)	349 in J'burg 302 in Tambo	J'burg: 80% < 5 y 5 y < = 15% < 15 y 3% > = 15 y Tambo: 41% < 5 y 5 y < = 49% < 15 y 8% > = 15 y	N/A	J'burg: Adm Cov 102% Tambo: Adm Cov: 90%	J'burg: 47.4% Tambo: 29.7%

Tanzania [31]	1999, 2001	July 06-Jan 07	Dar-Es-Salaam (2.5 M - 880000 < 14 y)	1533	Before response: 8% < 6 m 6 m = < 60% < 15 y 32% > = 15 y	29.3	84%	N/A

Sudan [26,27]	1998-2008	Mar to Jun 04	Darfur Region 2607082 Accessible 2170985	3 o/b: (West Darfur)01/03 to 27/04: 48 cases (North Darfur)27/03 to 16/06: 521 (West Darfur)01/04 to 03/06: 142	58% < 5 y	N/A	N/A	N/A

**EUROPE**								

Albania [32]	1999	04/99-06/99	442000 refugees from Kosovo	80	43% > = 15 y	N/A		unknown


*Abbreviations contained in the body of the table: N/A = not available, o/b = outbreak, w = week, m = month and y = year VE = reported vaccine effectiveness

**Table 2:** Mass vaccination response details *

Region/ Country	Time to response**	Target Area	Target Age	Doses/ Coverage	Author's Reported Impact	Documented Impact (authors' assessment)
**AMERICAS**						

Bolivia	MV1: 1998 4 m after 1^st ^case	Nonselective Nationwide	6 m -5 y	85%	Persistent cases	Epidemic ended after multiple immunization activities
		
	MV2: 1999	house-to-house Nationwide	6 m - 4 y + 6 m - 14 y in 2 dpts	98%	Persistent cases but decreased over time	
		
	MV3: 2000	House-to-house in high risk municipalities	N/A	N/A	N/A	
		
	MV4: 2002	House to house	6 m - 4 y	95%	Transmission stopped	

Haiti	MV1: < 4 w after 1^st ^case	Nonselective Provincial city	6 m - 14 y	95%	No cases in city within 2 w of end of campaign; spread to rest of island	Epidemic ended after multiple immunization activities
	MV2: N/A	Departments	6 m - 14 y	65 - 95%	No cases after early August in department	
		
	MV3: 5-9/00	Port-au-Prince	6 m - 14 y	82%		
		
	MV4: 11/00-1/01	Port-au-Prince neighborhood	6 m - 14 y	80 - 90%	Reduced number of cases island-wide	
		
	MV5: 9-12/01	Nationwide	N/A	> 85%	Measles transmission interrupted	

Colombia	Various	door to door vaccination in high risk municipalities	6 m-5 y	N/A	N/A but editorial suggests proactive response averted large outbreak	Compared to outbreak in neighboring Venezuela, prompt, door to door targeted vaccination and surveillance may have prevented a large outbreak in a country where EPI is limited by long term conflict

**ASIA**						

Afghanistan	12/2001-5/2002	Nonselective, Central region districts and returning refugees in catchment area. Revaccination in districts with low coverage	6 m-12 y	77% (62-90%) by May 2002 63-92% by December 2002	Impact on incidence not assessed. Campaign achieved high coverage despite many obstacles. Authors recommend vaccinating extended age groups in complex emergencies.	Unable to assess impact from data provided, but from WHO records measles incidence decreased dramatically for next 2 years.

India	Soon after flood began	Flood area, areas of congregation then cut-off villages	6 m to 14 y Catch-up	75% Catch-up:60%	Qualitative analysis on the vaccination in multiple stages. Initial one prevented large scale measles o/b and death, later stages contained smaller o/b and high mortality was prevented with a joint surveillance system	Insufficient data

India	Dec 29, 04 to Jan 9, 05	Non-selective, 58 villages in Namil-Tadu district, Eastern India	6 m to 60 m No catch-up	117.2%	Qualitative analysis transmission continued despite vaccine coverage and was unrelated to tsunami. Target age was too restrictive, recommendation to vaccinate children up to 14 years during complex emergencies like tsunami.	Insufficient data

Sri Lanka	N/A	Nonselective Refugee camps, welfare centers, preschools, & slums	Children " < 10 y"	N/A	N/A	Not clear

**AFRICA**						

Niger	Outreach services in some health centers	N/A	N/A	N/A	Impact not specified but authors discuss the need to include older than 5 y children in vaccination campaigns due to high CFR in this group.	Insufficient information to determine impact

Tanzania	Epidemic started in March, ORI were in April, June and August in 3 camps	Nonselective, refugee camps.	ORI: 6 m-5 y. But new arrivals 6 m-15 y are routinely vaccinated	N/A	6 m-5 y campaign prevented cases and deaths, but to halt transmission, campaigns targeting a wider age group would have been more effective	May have influenced epidemic. given large proportion of cases in older age groups, vaccinating up to age 15 early in the epidemic would have likely shortened the duration of the outbreaks.

Ethiopia	Within 1 month	Nonselective	9 m -5 y		Despite ORI in February measles cases continued to be reported in the district including among vaccinated. Recommend extending vaccinated age group to 12-15 y in acute emergencies. Epidemic was not halted until August when a vaccination campaign with grater coverage and efficacy implemented	The authors calculate low coverage and poor efficacy of vaccine in February campaign. These alone could have allowed outbreak to continue, but including a wider age range for vaccination may have been useful in containing the outbreak. No age breakdown of cases available.

Mozambique	Varied reactive SIAs	Nonselective, targeted urban (province capitals)	9 m-4 y		Measles campaigns had limited impact. Recommend increasing target age group and including rural areas linked to cities via transport routes.	Campaigns may have had some impact, as noted by reduced caseload in subsequent years. Targeting a wider age group in catch up and outbreak campaigns could have had greater impact.

Niger	Wk 24 after o/b	LQAS selection, 46 lots of 65 children	6 m - 5 y	Other SIAs after the survey: 99%	SIA are a first response to reinforcement of routine immunization activities (children under 5)	CFR = 3.3% (global o/b) No data otherwise

Nigeria	Wk 18 after o/b	Non-selective	6 m - 5 y	Other SIAs after the survey: 80%	same	

Chad	Wk 22 after o/b	Non-selective	6 m - 5 y	Other SIAs after the survey: 96%	same	

South Africa	Jan 04	Non-selective	6 m to 14 y Catch-up: 9 m-4 y	Catch-up: 86%	Importance of maintaining high immunity by means of routine immunization to prevent transmission following importation of the virus	N/A

Tanzania	11 wks after o/b	Non-selective	6 m to 14 y	882789 doses given Administrative: 100% Measured: 66%	Measles incidence declined in the targeted age group	Incidence would have been high in the target group without intervention

Sudan	06/05/04	North Darfur only	9 m - 15 y	93% of the accessible pop 77% of the global	The restricted access to population and the low coverage explains that measles cases still occurred after the vaccination campaign.	North Darfur: CFR = 17% West Darfur: CFR = 14% Similar results to other studies in comparable situations

**EUROPE**						

Albania	2 wks after o/b	Only two districts (Kukes and Has)	6 m - 5 y	90%	Surveillance system allowed for early epidemic detection	N/A

* Abbreviations contained in the body of the table: N/A = not available, d = day, w = week, m = month, y = year, o/b = outbreak, popn = population. For references of reports, see Table 1.

** In some cases, multiple rounds of vaccination were conducted. In this table, each round is designated by a number (ex, MV1).

†Selective indicates that only children without evidence of vaccination were targeted; nonselective indicates that all children regardless of vaccination status were targeted

In the Americas [9-12], there were no reports of preventive mass vaccination campaigns during the acute phase of a humanitarian emergency, but several reports of outbreak response immunization (ORI). An outbreak in Bolivia beginning in 1998 affected the country nationwide. A nationwide non-selective vaccination campaign, where children irrespective of their vaccination status are eligible for vaccination, was implemented four months after the first case was reported targeting children 6 m to 5 years with reported 85% coverage obtained in this age group. The following year house-to-house campaigns were performed in two departments of the country and in high-risk municipalities. In 2002, a house-to-house campaign was performed nationwide targeting children 6 m to 4 years with a reported 95% coverage and halt in transmission [9].

Similarly, in Haiti, cases were reported in Gonaives beginning on March 8, 2000. A non-selective mass vaccination campaign (single visit, house-to house) targeting children 6 m to 14 years was implemented at the end of April, 2000 with reported 95% coverage. The last case in Gonaives was reported on May 3, 2000. Subsequent campaigns were repeated in Artibonite, Port-au-Prince and Delmas after cases were reported there [10]. In Colombia, an epidemic in 2002 affected approximately one third of the country and a vaccination response was implemented door-to-door targeting children 6 months to 5 years in high risk areas. The authors posit that the prompt, although specific details of the delay are not given, door to door vaccination and surveillance may have prevented an even larger outbreak in a Colombia where routine services were limited by long-term conflict [11,12].

Reports from Asia include two non-selective mass vaccination interventions in response to natural disasters in India [13-20]. One response entailed the preventive vaccination of children in flooded areas of Bihar, where high population density and subsequent poor access to care placed the population at high risk. Non-selective vaccination of children 6 months to 14 years achieved an estimated 75% coverage. A total of 1811 measles cases were reported but there is insufficient data presented to determine the potential impact of this intervention, although the authors' qualitative analysis suggest that the campaign prevented a larger scale outbreak [13].

The second report from India describes the emergency response to the Indian Ocean earthquake and tsunami of 2004. Non-selective preventive mass vaccination for children 6 to 60 months was conducted in 58 villages of Tamil Nadu province, where one-dose measles coverage was reported to exceed 95%, beginning December 29, 2004, four days after the tsunami. A cluster of measles cases was subsequently reported in a tsunami affected area on December 30 with cases reported in non-tsunami affected areas of the province soon after. Although the overall scale of the outbreak was small (n = 101), the authors conclude that the target age range of the preventive vaccination was too restrictive as more than half of measles occurred in children between 5 and 15 years cases in both tsunami-affected villages (56.3%) and non-tsunami affected villages (60%) [14].

Two additional reports describe interventions in refugee populations [15-17]. In Afghanistan, following the fall of the Taliban, an influx of approximately 2 million refugees returning from Pakistan and other neighboring countries was anticipated in early 2002. In response, non-selective vaccination of children 6 months to 12 years was conducted throughout 2002 reaching 82%-96% of the target population by the end of 2002 [18]. The campaign initially targeted high-risk districts and cities with the largest number of susceptible children, and subsequently the most remote and inaccessible villages. A follow-up campaign was conducted in 2003, targeting children aged between 9 and 59 months. It is important to note that this campaign was prompted by the fact that an epidemic had occurred in 2001 affecting at least 7 of the 30 provinces in Afghanistan. Difficult access due to snow and mined roads and insecurity left many districts without heath services. The actual scale and scope of the 2001 epidemic is difficult to estimate, but a total of 8,762 cases were reported through the nationwide surveillance system, of which 33% of cases (n = 8762) occurred in children 5 to 12 years.

In Sri Lanka, a measles epidemic with a suspected 15,250 cases between October 1999 and June 2000 was reported [19]. The outbreak began in Colombo and progressed to becoming countrywide. Response included actively searching for and vaccinating children under the age of 10 years at the local level who did not report previous vaccination. Non-selective vaccination in "welfare centers, refugee camps, preschools, and urban slums" was also conducted without specifying the age range or whether all locations were included. The authors report that they "specifically chose not to implement outbreak response immunization as the WHO recommends such activity only under specific conditions such as refugee camps, military barracks or closed communities." The authors provide insufficient information with which to assess the potential impact of the intervention, but it is important to note that of the 3728 measles cases with sufficient detail, 40% reported having been vaccinated previously and 69.4% occurred in children over 10 years.

In the African region [21-31], two reports describe vaccination interventions in response to the nationwide epidemic in Niger in 2003-2004, where 50,138 cases were reported. A reactive campaign in the capital Niamey (n = 10,080 cases), targeted children 6 months to 5 years, 5 months after cases were reported [21]. In Mirrah District, Niger, outbreak response vaccination was restricted to outreach vaccination services in some health centers, although the extent of these efforts was not well documented [22]. The results of a retrospective household survey found two-thirds of case patients were under age 5 and 90% under the age of 10. The author's remark on the need to include children older than 5 years in vaccination activities as this may prevent deaths in infants who acquired measles from older children and also prevent deaths in older age groups, the rationale for the SPHERE recommendations. Mortality was inversely associated with the age of case patients, with the highest CFR in children under 12 months (15.7%; n = 13/83); followed by children 12-59 months (11.5%, n = 64/558); then children aged 5-14 years (5.4% (n = 14/259). In the same region, epidemics in Nigeria and Chad also occurred [23]. There was no vaccination response to the epidemic in Nigeria. A non-selective campaign targeting children 6 months to 5 years, four months after cases were reported, was implemented in N'djamena, Chad in 2005. Although subsequent SIAs in Niger, Nigeria and Chad reported obtaining high coverage among the target population, outbreaks continue to be reported in this region.

In Tanzania, a report on an outbreak among Burundian refugees in four camps noted 31% of cases were between 6 and 15 years [24]. A non-selective response targeting children 6 months to 5 years, initiated between one and five months after cases were first reported in each of the four camps reduced cases and deaths, however, the authors conclude that it would have been more effective to target a wider age range to halt transmission. A report on a measles epidemic in Gode, Ethiopia came to similar conclusion recommending that a wider age range than the 9 months to 5 years targeted in the response, which although prompt, could have contained the outbreak [25]. The authors further note the poor coverage achieved by the intervention and potentially poor vaccine efficacy due to presumed problems in the cold chain.

In Darfur, Sudan, although cases were reported throughout the Darfur region, non-selective vaccination targeting children 9 months to 15 years was conducted only in North Darfur, reaching a reported 93% of the accessible population, but an estimated 77% of the total target population [26,27]. Measles cases continued to occur after the intervention. The authors report difficulties accessing a population that was continually moving to avoid violence with the repercussion that new returnees to the camps were not vaccinated.

One report from Europe describes vaccination interventions in refugee populations [32]. In Albania, an epidemic response was initiated only two weeks after a measles outbreak began among Kosovar refugees in 1999. The surveillance system allowed for early detection of the outbreak and a non-selective campaign for children 6 months to 5 years was implemented. An estimated 43% of the 80 cases were in persons older than 15 years.

## Discussion

In humanitarian emergencies, long-term disruption of routine vaccination programs leave large populations unvaccinated, thereby increasing the risk of measles outbreaks. Poor access to health services, ongoing displacement and population movements further limit the ability to obtain high vaccination coverage and increase mortality. Outbreaks of measles continue to occur in humanitarian emergencies and while routine programs are crucial, additional vaccination activities are vital to ensure population protection to reduce morbidity and mortality.

**Figure 1:**
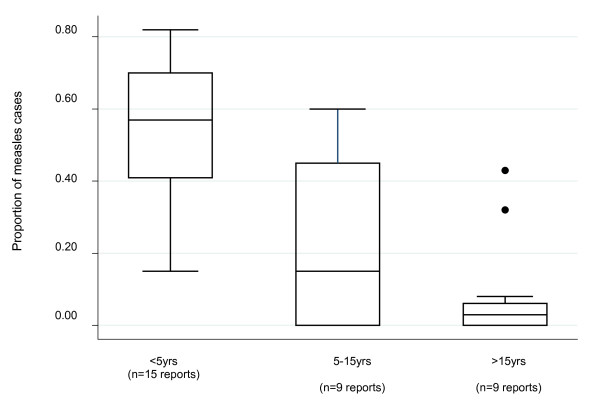
**Proportion of measles cases by age group in reports including these data from 15 countries, 1998-2010**.

Of the few well-documented epidemic descriptions in humanitarian emergencies, the age range of cases is not limited to under 5 year olds. Combining all data, both from preventive and outbreak response interventions, about 59% of cases in reports with sufficient data reviewed here remain in children under 5, 18% in 5-15 and 2% above 15 years (Figure 1). In instances where interventions targeted a reduced age range, several reports concluded that the age range should have been extended to 15 years, given that a significant proportion of cases occurred beyond 5 years of age. Non-selective mass vaccination of children 6 months to 15 years remains the most prudent option for reducing measles morbidity and mortality in emergencies. In some cases, vaccination of age groups greater than 15 years may need to be considered based on a risk assessment of the area including whether the country has a mortality reduction or elimination goal. Recent epidemics in Burkina Faso and Malawi, although not in the context of a humanitarian emergency, reported more than one third of cases over the age of 15 years. In humanitarian emergencies, particularly in protracted crises, routine services may be compromised for many years and thus older age groups may not have been routinely vaccinated. Older age groups continue to be left out as the routine program targets children under 5, again highlighting the importance of mass campaigns and increasing the target age group for mass campaigns to 15 years.

However, we recognize that in some cases, target age groups may need to be reduced due to lack of medically trained staff, extreme security situations or limited vaccine supplies. The current ongoing epidemic in the Democratic Republic of Congo, spanning a large geographic area and population presents a serious challenge in terms of a rapid response and in this case if it is only possible to vaccinate a portion of children at risk, children 6-59 months should be prioritized. We recognize that extenuating circumstances may necessitate allocation of scare resources and less optimal strategies put in place.

While mass vaccination for measles in humanitarian emergencies remains necessary, the best and most cost-effective approach is to prevent epidemics entirely by ensuring high first dose routine vaccination coverage and a second opportunity for measles vaccination for all children. Humanitarian emergencies are overlaid onto contexts with differing level of pre-existing population immunity, which influence the risk of an outbreak. Countries in the Americas, where measles has been eliminated due to a high quality and sustained effort, have smaller scale epidemics occurring in a setting where routine vaccination remains the core of the control effort. Conversely, larger scale epidemics in countries like Afghanistan, where routine services have been interrupted for more than 20 years and insecurity curtails both preventive and reactive vaccination, have continued for long periods. There is a critical need to consider the epidemiology of measles within the context of the crisis in order to provide an adapted response.

This review has important limitations. First, we restricted our review to the published literature. Although we did search the grey literature through the collection of international agency and NGO documentation, conclusions from the grey literature are severely limited. Reports and databases focus often on delivery and rather than on an epidemiologic analysis of the intervention. This is due largely to the fact that formal documentation of emergency response is not a part of the standard operating procedure of many emergency organizations. It is not a routine part of the professional culture, and when reports exist, they relate to a single organization's response and are often for internal use or limited distribution. By relying only on the published literature this review suffers from a clear publication bias. Reports relating to responses in humanitarian emergencies are rare as the necessary and important aspects of publication are not often met (ethical approval, study protocol, logistic constraints and poor awareness of the publication process) and documentation of events may be low on the list of priorities in often overwhelming situations where the primary goal is to deliver and provide aid to a population in order to reduce mortality and morbidity. Nevertheless, we chose to review the published literature, as however scanty, it still remains the reference for evidence-based guidance. An additional limitation to focusing on the published literature that there are scarce reports of reactive or preventive vaccination campaigns in emergency settings where no measles outbreak occurred. This is an important part of evaluating the impact of current recommendations; however, such situations are even less likely to be published.

A third and related limitation is the choice of our definition for emergencies. We chose to use the definition of countries applying for a CAP or Flash appeal during the period of our review. We also included countries that had ever applied for assistance during the time period, whether this coincided with the reported epidemic or not. As a result, humanitarian emergencies were not included if they occurred in a country that did not apply for CAP or Flash appeals.

As the landscape of emergencies changes, epidemics in countries not undergoing armed conflict or natural disaster, but rather political instability, dire poverty and displacement from trans-boundary or regional conflicts become increasingly frequent sites for emergency interventions. Displaced persons, whether escaping violence or seeking employment and assistance, increasingly seek refuge in cities, as reflected by the number of large urban outbreaks included in this review. Alternately, rural and remote areas with dispersed populations may become a more frequent site for intervention as care provision and maintaining sufficient vaccination coverage in these areas is difficult. Responding to the risk of a measles outbreak in rural areas bears closer similarities to an emergency response than in a stable setting. Measles outbreak responses in humanitarian emergencies are predominantly campaign-based, the population denominator is often unknown or unreliable and the response is often done in coordination or partnership with UN agencies and disaster relief agencies. This is contrasted with a stable setting where the response may be undertaken predominantly through fixed sites and the national infrastructure.

Perhaps the most important result of this review is to highlight the need for improved documentation of mass vaccination campaigns and measles epidemics in emergencies. This baseline review of documented interventions, meeting a relatively broad criteria, suggest that further efforts are needed to encourage formal documentation and evaluation of emergency responses including comparison of the cost-effectiveness and cost-benefits of different vaccination strategies. Although guidance for mass measles vaccination in humanitarian emergencies is not controversial, implementation of an immediate preventive response remains challenging.

## Conclusions

Measles outbreaks continue to occur in humanitarian emergencies due to low levels of pre-existing population immunity. According to available published information, cases continue to occur in children over age 5. Preventing cases in older age groups may prevent younger children from becoming infected and reduce mortality in both younger and older age groups. As measles vaccination coverage increases globally, outbreaks have become less frequent and the age distribution of cases has shifted towards older age groups. Hence there is a need to consider the context of the emergency and make a quick assessment of the likely immunity profile among the affected population, taking into account the year in which routine measles vaccination was introduced into the country, when supplementary vaccination activities occurred, and the likely vaccination history (and hence immunity level) of each age group affected by the emergency. As this information may often be lacking or incomplete, based on recent experience, the existing SPHERE recommendation to vaccinate all children 6 months to 15 years remains sound public health policy.

## Competing interests

The authors declare that they have no competing interests.

## Authors' contributions

RFG drafted the manuscript. All authors participated in the design of the study and coordination and helped to draft the manuscript. All authors read and approved the final manuscript.
